# Ethyl 4-[2-(3,5-dimethyl-4-oxo-2,6-diphenyl­piperidin-1-yl)-2-oxoeth­yl]piperazine-1-carboxyl­ate

**DOI:** 10.1107/S1600536811003412

**Published:** 2011-02-02

**Authors:** Mannangatty Rani, Rajamanickam Ramachandran, Senthamaraikannan Kabilan, Yeon Tae Jeong

**Affiliations:** a Department of Chemistry, Annamalai University, Annamalai Nagar 608 002, Tamil Nadu, India; b Department of Image Science and Engineering, Pukyong National University, Busan 608-737, Republic of Korea

## Abstract

In the title compound, C_28_H_35_N_3_O_4_, the piperidine ring adopts a boat conformation while the piperazine ring adopts a chair conformation with an equatorial orientation of the phenyl groups. The dihedral angle between the mean planes of the benzene rings is 74.14 (8)°. The mol­ecular conformation is stabilized by a weak intra­molecular C—H⋯N inter­action and the crystal packing is stabilized by weak inter­molecular C—H⋯O inter­actions.

## Related literature

For the biological activity of related structures, see: El-subbagh *et al.* (2000 ▶); Emami *et al.* (2006 ▶); Foroumadi *et al.* (2007 ▶); Katritzky & Fan (1990 ▶); Mobio *et al.* (1989 ▶). For geometrical analysis, see: Cremer & Pople (1975 ▶); Emami *et al.* (2006 ▶); Foroumadi *et al.* (2007 ▶); Nardelli (1983 ▶).
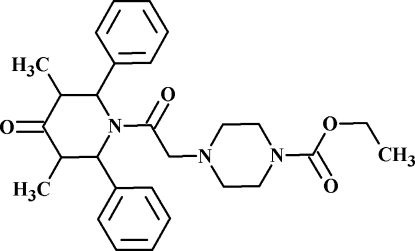


## Experimental

### 

#### Crystal data

C_28_H_35_N_3_O_4_
*M* *_r_* = 477.59
Monoclinic, 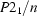

*a* = 10.9073 (3) Å
*b* = 19.1940 (6) Å
*c* = 12.2246 (3) Å
β = 91.809 (2)°
*V* = 2558.00 (12) Å^3^
*Z* = 4
Mo *K*α radiation
μ = 0.08 mm^−1^
*T* = 293 K
0.30 × 0.20 × 0.20 mm

#### Data collection

Bruker Kappa APEXII CCD diffractometer
Absorption correction: multi-scan (*SADABS*; Bruker, 1999 ▶) *T*_min_ = 0.975, *T*_max_ = 0.984
31692 measured reflections
6941 independent reflections
4703 reflections with *I* > 2σ(*I*)
*R*_int_ = 0.029

#### Refinement

*R*[*F* ^2^ > 2σ(*F* ^2^)] = 0.047
*wR*(*F* ^2^) = 0.142
*S* = 1.01
6941 reflections
316 parameters
H-atom parameters constrained
Δρ_max_ = 0.38 e Å^−3^
Δρ_min_ = −0.18 e Å^−3^

### 

Data collection: *APEX2* (Bruker, 2004 ▶); cell refinement: *APEX2* and *SAINT* (Bruker, 2004 ▶); data reduction: *SAINT* and *XPREP* (Bruker, 2004 ▶); program(s) used to solve structure: *SIR92* (Altomare *et al.*, 1993) ▶; program(s) used to refine structure: *SHELXL97* (Sheldrick, 2008 ▶); molecular graphics: *ORTEP-3* (Farrugia, 1997 ▶); software used to prepare material for publication: *SHELXL97*.

## Supplementary Material

Crystal structure: contains datablocks global, I. DOI: 10.1107/S1600536811003412/zq2086sup1.cif

Structure factors: contains datablocks I. DOI: 10.1107/S1600536811003412/zq2086Isup2.hkl

Additional supplementary materials:  crystallographic information; 3D view; checkCIF report

## Figures and Tables

**Table 1:** Hydrogen-bond geometry (Å, °)

*D*—H⋯*A*	*D*—H	H⋯*A*	*D*⋯*A*	*D*—H⋯*A*
C5—H5⋯N2	0.98	2.51	3.1788 (17)	125
C8—H8⋯O2^i^	0.93	2.54	3.4406 (19)	162
C24—H24*A*⋯O1^ii^	0.97	2.56	3.520 (2)	169

Symmetry codes: (i) 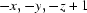; (ii) 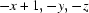.

**Fig. 1.**
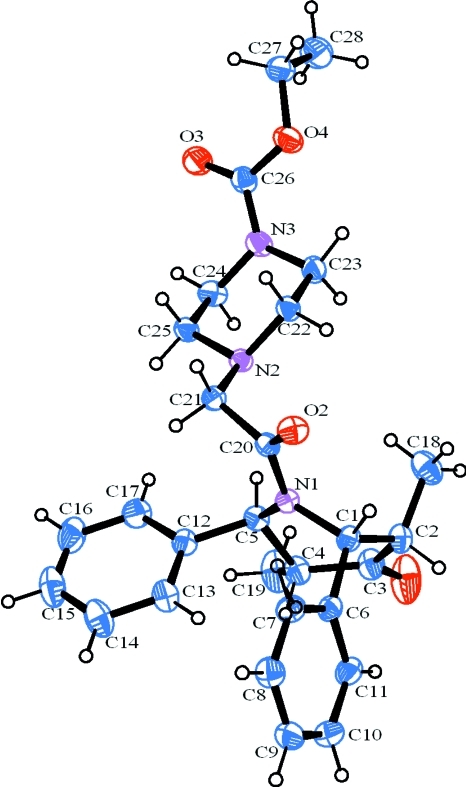
A view of the molecular structure of the title compound. Displacement ellipsoids are drawn at the 30% probability level.

## supplementary crystallographic information

### Comment

Several interesting investigations have been carried out with piperidine based heterocyclic compounds and these compounds were found to exhibit numerous pharmacological properties and biological activities such as anticancer, antimicrobial, anti-inflammatory, antiviral, antimalarial and anesthetics (El-subbagh *et al.*, 2000; Mobio *et al.*, 1989; Katritzky & Fan, 1990). Similarly, some compounds containing piperazine are used as antibiotic drugs, *e.g.*, Norfloxacin, Ciprofloxacin, Enoxacin, Ofloxacin and Levofloxacine (Emami *et al.*, 2006; Foroumadi *et al.*, 2007).

In the title compound, C_28_H_35_N_3_O_4_, the piperidine ring adopts a boat conformation. The corresponding puckering parameters (Cremer & Pople, 1975) and smallest displacement asymmetry parameters (Nardelli, 1983) are q_1_ = 0.6111 (15) Å, q_2_ = -0.0839 (15) Å, Q_T _= 0.6168 (15) Å, and θ = 97.81 (14) °. Unlike, the piperazine ring adopts a chair conformation with q_1_ = 0.0367 (15), q_2_ = 0.5606 (15) Å, Q_T _= 0.5618 (15) Å and θ = 3.75 (15)°. The phenyl groups are orientated to the same side of the piperazine ring. The dihedral angle between the mean planes of the benzene rings is 74.14 (8)°. The molecular conformation is stabilized by a weak intramolecular C5-H5···N2 interaction and the crystal packing by the weak intermolecular C8-H8···O2^i^ and C24-H24···O1^ii^ interactions [Table 1; symmetry codes: (i) -*x*, -*y*, -*z*+1; (ii) -*x*+1, -*y*, -*z*].

### Experimental

A mixture of *N*-chloroacetyl-3,5-dimethyl-2,6-diphenylpiperidin-4-one (0.005 mol), triethylamine (0.01 mol) and *N*-ethoxycarbonylpiperazine (0.005 mol) in toluene were refluxed for about 6–8 h. After the completion of reaction, excess of solvent was removed under reduced pressure. The obtained residue was column chromatographed on silica gel using benzene:ethyl acetate (2:1) mixture as an eluent which afforded the title compound in good yield. Colourless crystals were grown by slow evaporation method using ethanol as solvent.

### Refinement

H atoms were positioned and refined using a riding model, with aromatic C—H = 0.93 Å, methine C—H = 0.98 Å, methylene C—H = 0.97 Å and methyl C—H = 0.96 Å. The displacement parameters were set to *U*_iso_(H) = 1.5*U*_eq_(C) for the methyl H atoms and to *U*_iso_(H) = 1.2*U*_eq_(C) for the other H atoms.

### Crystal data

**Table:**

C_28_H_35_N_3_O_4_	*Z* = 4
*M**_r_* = 477.59	*F*(000) = 1024
Monoclinic, *P*2_1_/*n*	*D*_x_ = 1.240 Mg m^−^^3^
Hall symbol: -P 2yn	Mo *K*α radiation, λ = 0.71073 Å
*a* = 10.9073 (3) Å	θ = 2.0–29.2°
*b* = 19.1940 (6) Å	µ = 0.08 mm^−^^1^
*c* = 12.2246 (3) Å	*T* = 293 K
β = 91.809 (2)°	Prism, colourless
*V* = 2558.00 (12) Å^3^	0.30 × 0.20 × 0.20 mm

### Data collection

**Table:**

Bruker Kappa APEXII CCD diffractometer	6941 independent reflections
Radiation source: fine-focus sealed tube	4703 reflections with *I* > 2σ(*I*)
graphite	*R*_int_ = 0.029
ω and φ scans	θ_max_ = 29.2°, θ_min_ = 2.0°
Absorption correction: multi-scan (*SADABS*; Bruker, 1999)	*h* = −14→14
*T*_min_ = 0.975, *T*_max_ = 0.984	*k* = −26→26
31692 measured reflections	*l* = −16→16

### Refinement

**Table:**

Refinement on *F*^2^	Primary atom site location: structure-invariant direct methods
Least-squares matrix: full	Secondary atom site location: difference Fourier map
*R*[*F*^2^ > 2σ(*F*^2^)] = 0.047	Hydrogen site location: inferred from neighbouring sites
*wR*(*F*^2^) = 0.142	H-atom parameters constrained
*S* = 1.01	*w* = 1/[σ^2^(*F*_o_^2^) + (0.0676*P*)^2^ + 0.4844*P*] where *P* = (*F*_o_^2^ + 2*F*_c_^2^)/3
6941 reflections	(Δ/σ)_max_ < 0.001
316 parameters	Δρ_max_ = 0.38 e Å^−^^3^
0 restraints	Δρ_min_ = −0.18 e Å^−^^3^

### Special details

**Table:**

Geometry. All e.s.d.'s (except the e.s.d. in the dihedral angle between two l.s. planes) are estimated using the full covariance matrix. The cell e.s.d.'s are taken into account individually in the estimation of e.s.d.'s in distances, angles and torsion angles; correlations between e.s.d.'s in cell parameters are only used when they are defined by crystal symmetry. An approximate (isotropic) treatment of cell e.s.d.'s is used for estimating e.s.d.'s involving l.s. planes.
Refinement. Refinement of *F*^2^ against ALL reflections. The weighted *R*-factor *wR* and goodness of fit *S* are based on *F*^2^, conventional *R*-factors *R* are based on *F*, with *F* set to zero for negative *F*^2^. The threshold expression of *F*^2^ > σ(*F*^2^) is used only for calculating *R*-factors(gt) *etc*. and is not relevant to the choice of reflections for refinement. *R*-factors based on *F*^2^ are statistically about twice as large as those based on *F*, and *R*- factors based on ALL data will be even larger.

### Fractional atomic coordinates and isotropic or equivalent isotropic displacement parameters (Å^2^)

**Table:**

	*x*	*y*	*z*	*U*_iso_*/*U*_eq_	
O2	0.30276 (10)	0.02315 (6)	0.45786 (8)	0.0474 (3)	
N1	0.27565 (10)	0.01252 (6)	0.27464 (8)	0.0346 (2)	
O3	0.83211 (11)	0.26851 (6)	0.33044 (11)	0.0674 (4)	
O4	0.87576 (10)	0.17829 (6)	0.44239 (10)	0.0594 (3)	
N2	0.48203 (10)	0.12188 (6)	0.31107 (9)	0.0354 (2)	
N3	0.72912 (11)	0.16692 (7)	0.31251 (11)	0.0469 (3)	
O1	0.36909 (18)	−0.11603 (8)	0.02744 (11)	0.1013 (6)	
C21	0.35944 (12)	0.12158 (7)	0.35490 (11)	0.0385 (3)	
H21A	0.3047	0.1482	0.3068	0.046*	
H21B	0.3618	0.1441	0.4260	0.046*	
C20	0.30991 (11)	0.04850 (7)	0.36633 (10)	0.0346 (3)	
C1	0.24946 (13)	−0.06249 (7)	0.28730 (11)	0.0379 (3)	
H1	0.2739	−0.0741	0.3630	0.045*	
C23	0.69961 (13)	0.09713 (8)	0.34954 (14)	0.0481 (4)	
H23A	0.7577	0.0831	0.4071	0.058*	
H23B	0.7059	0.0646	0.2893	0.058*	
C6	0.11293 (13)	−0.07761 (7)	0.27691 (11)	0.0391 (3)	
C2	0.33470 (14)	−0.10402 (8)	0.21546 (12)	0.0458 (3)	
H2	0.3107	−0.1532	0.2182	0.055*	
C7	0.03802 (14)	−0.04948 (8)	0.35541 (12)	0.0454 (3)	
H7	0.0729	−0.0228	0.4118	0.054*	
C12	0.18481 (14)	0.09997 (7)	0.14324 (11)	0.0414 (3)	
C5	0.28059 (13)	0.04335 (7)	0.16348 (10)	0.0380 (3)	
H5	0.3615	0.0648	0.1569	0.046*	
C4	0.26648 (15)	−0.01210 (8)	0.07262 (11)	0.0473 (3)	
H4	0.1785	−0.0218	0.0642	0.057*	
C22	0.57133 (12)	0.09542 (7)	0.39208 (12)	0.0405 (3)	
H22A	0.5503	0.0479	0.4110	0.049*	
H22B	0.5682	0.1234	0.4580	0.049*	
C11	0.05813 (15)	−0.11776 (8)	0.19519 (13)	0.0498 (4)	
H11	0.1063	−0.1380	0.1424	0.060*	
C3	0.32775 (16)	−0.08057 (9)	0.09817 (13)	0.0540 (4)	
C13	0.06182 (15)	0.08840 (9)	0.15817 (13)	0.0518 (4)	
H13	0.0359	0.0452	0.1832	0.062*	
C25	0.51477 (13)	0.19283 (7)	0.28016 (12)	0.0434 (3)	
H25A	0.5134	0.2228	0.3440	0.052*	
H25B	0.4549	0.2104	0.2266	0.052*	
C24	0.64026 (14)	0.19466 (9)	0.23281 (12)	0.0490 (4)	
H24A	0.6408	0.1670	0.1664	0.059*	
H24B	0.6616	0.2422	0.2145	0.059*	
C8	−0.08692 (14)	−0.06037 (9)	0.35137 (14)	0.0516 (4)	
H8	−0.1355	−0.0410	0.4046	0.062*	
C9	−0.13964 (15)	−0.09967 (9)	0.26901 (15)	0.0560 (4)	
H9	−0.2239	−0.1070	0.2660	0.067*	
C17	0.21955 (18)	0.16440 (9)	0.10412 (14)	0.0577 (4)	
H17	0.3017	0.1731	0.0913	0.069*	
C26	0.81377 (13)	0.20954 (8)	0.35904 (13)	0.0470 (3)	
C10	−0.06694 (16)	−0.12810 (9)	0.19096 (15)	0.0590 (4)	
H10	−0.1025	−0.1546	0.1347	0.071*	
C18	0.46803 (16)	−0.09787 (11)	0.25605 (16)	0.0661 (5)	
H18A	0.4750	−0.1126	0.3310	0.099*	
H18B	0.4943	−0.0503	0.2504	0.099*	
H18C	0.5187	−0.1269	0.2122	0.099*	
C27	0.97832 (14)	0.21660 (10)	0.49077 (15)	0.0575 (4)	
H27A	0.9891	0.2040	0.5673	0.069*	
H27B	0.9616	0.2662	0.4867	0.069*	
C16	0.1336 (2)	0.21594 (9)	0.08396 (15)	0.0719 (6)	
H16	0.1586	0.2593	0.0590	0.086*	
C14	−0.02354 (18)	0.14019 (11)	0.13642 (15)	0.0678 (5)	
H14	−0.1063	0.1315	0.1465	0.081*	
C15	0.0126 (2)	0.20387 (11)	0.10038 (16)	0.0750 (6)	
H15	−0.0450	0.2388	0.0871	0.090*	
C19	0.3060 (2)	0.01732 (12)	−0.03613 (14)	0.0801 (7)	
H19A	0.2654	0.0610	−0.0497	0.120*	
H19B	0.2845	−0.0148	−0.0937	0.120*	
H19C	0.3932	0.0244	−0.0336	0.120*	
C28	1.09184 (17)	0.20086 (12)	0.43263 (18)	0.0743 (5)	
H28A	1.1589	0.2266	0.4653	0.111*	
H28B	1.0813	0.2138	0.3571	0.111*	
H28C	1.1090	0.1519	0.4377	0.111*	

### Atomic displacement parameters (Å^2^)

**Table:**

	*U*^11^	*U*^22^	*U*^33^	*U*^12^	*U*^13^	*U*^23^
O2	0.0544 (6)	0.0553 (6)	0.0324 (5)	−0.0062 (5)	0.0018 (4)	0.0037 (4)
N1	0.0375 (6)	0.0359 (6)	0.0303 (5)	−0.0015 (4)	0.0027 (4)	0.0030 (4)
O3	0.0553 (7)	0.0542 (7)	0.0916 (9)	−0.0118 (5)	−0.0117 (6)	0.0157 (7)
O4	0.0454 (6)	0.0619 (7)	0.0702 (8)	−0.0039 (5)	−0.0110 (5)	0.0083 (6)
N2	0.0334 (5)	0.0359 (6)	0.0368 (6)	−0.0021 (4)	0.0000 (4)	0.0029 (4)
N3	0.0357 (6)	0.0506 (7)	0.0544 (7)	−0.0048 (5)	0.0009 (5)	0.0106 (6)
O1	0.1630 (17)	0.0893 (10)	0.0528 (8)	0.0586 (11)	0.0210 (9)	−0.0109 (7)
C21	0.0356 (7)	0.0385 (7)	0.0414 (7)	0.0010 (5)	0.0016 (5)	−0.0017 (6)
C20	0.0284 (6)	0.0408 (7)	0.0347 (7)	0.0021 (5)	0.0036 (5)	0.0009 (5)
C1	0.0429 (7)	0.0350 (7)	0.0359 (7)	0.0000 (5)	0.0037 (5)	0.0029 (5)
C23	0.0386 (7)	0.0415 (8)	0.0642 (10)	0.0019 (6)	0.0009 (7)	0.0053 (7)
C6	0.0447 (7)	0.0325 (6)	0.0402 (7)	−0.0026 (5)	0.0020 (6)	0.0034 (5)
C2	0.0509 (8)	0.0414 (7)	0.0454 (8)	0.0084 (6)	0.0059 (6)	0.0003 (6)
C7	0.0473 (8)	0.0467 (8)	0.0423 (8)	−0.0063 (6)	0.0044 (6)	−0.0023 (6)
C12	0.0502 (8)	0.0430 (7)	0.0308 (6)	0.0022 (6)	0.0003 (6)	0.0038 (5)
C5	0.0400 (7)	0.0436 (7)	0.0306 (6)	−0.0016 (6)	0.0037 (5)	0.0061 (5)
C4	0.0538 (9)	0.0560 (9)	0.0323 (7)	0.0098 (7)	0.0023 (6)	−0.0018 (6)
C22	0.0381 (7)	0.0391 (7)	0.0439 (7)	−0.0009 (5)	−0.0020 (6)	0.0079 (6)
C11	0.0549 (9)	0.0421 (8)	0.0525 (9)	−0.0010 (7)	0.0014 (7)	−0.0093 (7)
C3	0.0638 (10)	0.0560 (9)	0.0426 (8)	0.0128 (8)	0.0065 (7)	−0.0071 (7)
C13	0.0530 (9)	0.0537 (9)	0.0491 (9)	0.0088 (7)	0.0108 (7)	0.0091 (7)
C25	0.0416 (7)	0.0406 (7)	0.0475 (8)	−0.0032 (6)	−0.0077 (6)	0.0109 (6)
C24	0.0485 (8)	0.0568 (9)	0.0415 (8)	−0.0098 (7)	−0.0012 (6)	0.0114 (7)
C8	0.0465 (8)	0.0525 (9)	0.0563 (9)	0.0005 (7)	0.0090 (7)	0.0049 (7)
C9	0.0439 (8)	0.0526 (9)	0.0710 (11)	−0.0026 (7)	−0.0050 (8)	0.0072 (8)
C17	0.0710 (11)	0.0515 (9)	0.0501 (9)	−0.0067 (8)	−0.0075 (8)	0.0152 (7)
C26	0.0344 (7)	0.0501 (9)	0.0565 (9)	−0.0004 (6)	0.0049 (6)	0.0050 (7)
C10	0.0600 (10)	0.0494 (9)	0.0665 (11)	−0.0061 (7)	−0.0146 (8)	−0.0075 (8)
C18	0.0532 (10)	0.0853 (13)	0.0599 (10)	0.0208 (9)	0.0053 (8)	−0.0007 (9)
C27	0.0483 (9)	0.0631 (10)	0.0605 (10)	0.0028 (7)	−0.0062 (7)	−0.0116 (8)
C16	0.1107 (17)	0.0461 (10)	0.0582 (11)	0.0082 (10)	−0.0092 (11)	0.0140 (8)
C14	0.0632 (11)	0.0812 (13)	0.0598 (11)	0.0263 (10)	0.0130 (8)	0.0089 (10)
C15	0.0996 (16)	0.0673 (12)	0.0581 (11)	0.0372 (12)	0.0015 (11)	0.0078 (9)
C19	0.1185 (18)	0.0870 (14)	0.0359 (9)	0.0339 (13)	0.0186 (10)	0.0095 (9)
C28	0.0501 (10)	0.0823 (14)	0.0905 (14)	0.0029 (9)	0.0049 (9)	−0.0082 (11)

### Geometric parameters (Å, °)

**Table:**

O2—C20	1.2250 (15)	C4—H4	0.9800
N1—C20	1.3590 (17)	C22—H22A	0.9700
N1—C1	1.4768 (17)	C22—H22B	0.9700
N1—C5	1.4846 (16)	C11—C10	1.378 (2)
O3—C26	1.2032 (19)	C11—H11	0.9300
O4—C26	1.3460 (18)	C13—C14	1.382 (2)
O4—C27	1.4491 (19)	C13—H13	0.9300
N2—C21	1.4561 (17)	C25—C24	1.503 (2)
N2—C22	1.4583 (16)	C25—H25A	0.9700
N2—C25	1.4606 (17)	C25—H25B	0.9700
N3—C26	1.3461 (19)	C24—H24A	0.9700
N3—C23	1.4534 (19)	C24—H24B	0.9700
N3—C24	1.4535 (18)	C8—C9	1.370 (2)
O1—C3	1.1995 (19)	C8—H8	0.9300
C21—C20	1.5111 (19)	C9—C10	1.373 (3)
C21—H21A	0.9700	C9—H9	0.9300
C21—H21B	0.9700	C17—C16	1.380 (3)
C1—C6	1.519 (2)	C17—H17	0.9300
C1—C2	1.5240 (19)	C10—H10	0.9300
C1—H1	0.9800	C18—H18A	0.9600
C23—C22	1.508 (2)	C18—H18B	0.9600
C23—H23A	0.9700	C18—H18C	0.9600
C23—H23B	0.9700	C27—C28	1.478 (2)
C6—C11	1.383 (2)	C27—H27A	0.9700
C6—C7	1.389 (2)	C27—H27B	0.9700
C2—C3	1.502 (2)	C16—C15	1.362 (3)
C2—C18	1.526 (2)	C16—H16	0.9300
C2—H2	0.9800	C14—C15	1.362 (3)
C7—C8	1.378 (2)	C14—H14	0.9300
C7—H7	0.9300	C15—H15	0.9300
C12—C13	1.378 (2)	C19—H19A	0.9600
C12—C17	1.383 (2)	C19—H19B	0.9600
C12—C5	1.5224 (19)	C19—H19C	0.9600
C5—C4	1.542 (2)	C28—H28A	0.9600
C5—H5	0.9800	C28—H28B	0.9600
C4—C3	1.503 (2)	C28—H28C	0.9600
C4—C19	1.519 (2)		
			
C20—N1—C1	117.26 (10)	O1—C3—C2	120.61 (15)
C20—N1—C5	122.37 (11)	O1—C3—C4	121.42 (15)
C1—N1—C5	119.82 (10)	C2—C3—C4	117.97 (13)
C26—O4—C27	116.47 (13)	C12—C13—C14	120.76 (16)
C21—N2—C22	110.50 (10)	C12—C13—H13	119.6
C21—N2—C25	109.50 (10)	C14—C13—H13	119.6
C22—N2—C25	109.69 (10)	N2—C25—C24	110.76 (12)
C26—N3—C23	125.75 (13)	N2—C25—H25A	109.5
C26—N3—C24	119.64 (13)	C24—C25—H25A	109.5
C23—N3—C24	113.39 (12)	N2—C25—H25B	109.5
N2—C21—C20	111.89 (11)	C24—C25—H25B	109.5
N2—C21—H21A	109.2	H25A—C25—H25B	108.1
C20—C21—H21A	109.2	N3—C24—C25	109.23 (12)
N2—C21—H21B	109.2	N3—C24—H24A	109.8
C20—C21—H21B	109.2	C25—C24—H24A	109.8
H21A—C21—H21B	107.9	N3—C24—H24B	109.8
O2—C20—N1	121.85 (12)	C25—C24—H24B	109.8
O2—C20—C21	119.11 (12)	H24A—C24—H24B	108.3
N1—C20—C21	119.03 (11)	C9—C8—C7	120.10 (16)
N1—C1—C6	111.71 (10)	C9—C8—H8	120.0
N1—C1—C2	109.03 (11)	C7—C8—H8	120.0
C6—C1—C2	117.79 (12)	C8—C9—C10	119.43 (16)
N1—C1—H1	105.8	C8—C9—H9	120.3
C6—C1—H1	105.8	C10—C9—H9	120.3
C2—C1—H1	105.8	C16—C17—C12	120.73 (18)
N3—C23—C22	110.14 (12)	C16—C17—H17	119.6
N3—C23—H23A	109.6	C12—C17—H17	119.6
C22—C23—H23A	109.6	O3—C26—O4	123.71 (14)
N3—C23—H23B	109.6	O3—C26—N3	124.54 (14)
C22—C23—H23B	109.6	O4—C26—N3	111.75 (13)
H23A—C23—H23B	108.1	C9—C10—C11	120.64 (15)
C11—C6—C7	117.74 (14)	C9—C10—H10	119.7
C11—C6—C1	124.57 (13)	C11—C10—H10	119.7
C7—C6—C1	117.68 (12)	C2—C18—H18A	109.5
C3—C2—C1	112.33 (12)	C2—C18—H18B	109.5
C3—C2—C18	107.81 (14)	H18A—C18—H18B	109.5
C1—C2—C18	111.38 (13)	C2—C18—H18C	109.5
C3—C2—H2	108.4	H18A—C18—H18C	109.5
C1—C2—H2	108.4	H18B—C18—H18C	109.5
C18—C2—H2	108.4	O4—C27—C28	110.34 (15)
C8—C7—C6	121.26 (14)	O4—C27—H27A	109.6
C8—C7—H7	119.4	C28—C27—H27A	109.6
C6—C7—H7	119.4	O4—C27—H27B	109.6
C13—C12—C17	117.94 (14)	C28—C27—H27B	109.6
C13—C12—C5	121.97 (13)	H27A—C27—H27B	108.1
C17—C12—C5	120.03 (14)	C15—C16—C17	120.54 (18)
N1—C5—C12	112.88 (11)	C15—C16—H16	119.7
N1—C5—C4	112.24 (11)	C17—C16—H16	119.7
C12—C5—C4	108.85 (11)	C15—C14—C13	120.53 (19)
N1—C5—H5	107.5	C15—C14—H14	119.7
C12—C5—H5	107.5	C13—C14—H14	119.7
C4—C5—H5	107.5	C14—C15—C16	119.48 (17)
C3—C4—C19	111.72 (14)	C14—C15—H15	120.3
C3—C4—C5	114.84 (12)	C16—C15—H15	120.3
C19—C4—C5	110.43 (14)	C4—C19—H19A	109.5
C3—C4—H4	106.4	C4—C19—H19B	109.5
C19—C4—H4	106.4	H19A—C19—H19B	109.5
C5—C4—H4	106.4	C4—C19—H19C	109.5
N2—C22—C23	111.42 (12)	H19A—C19—H19C	109.5
N2—C22—H22A	109.3	H19B—C19—H19C	109.5
C23—C22—H22A	109.3	C27—C28—H28A	109.5
N2—C22—H22B	109.3	C27—C28—H28B	109.5
C23—C22—H22B	109.3	H28A—C28—H28B	109.5
H22A—C22—H22B	108.0	C27—C28—H28C	109.5
C10—C11—C6	120.82 (15)	H28A—C28—H28C	109.5
C10—C11—H11	119.6	H28B—C28—H28C	109.5
C6—C11—H11	119.6		
			
C22—N2—C21—C20	−70.96 (14)	C25—N2—C22—C23	−57.61 (15)
C25—N2—C21—C20	168.12 (11)	N3—C23—C22—N2	54.42 (16)
C1—N1—C20—O2	−10.29 (18)	C7—C6—C11—C10	1.3 (2)
C5—N1—C20—O2	178.25 (12)	C1—C6—C11—C10	−179.10 (14)
C1—N1—C20—C21	169.07 (11)	C1—C2—C3—O1	−164.94 (19)
C5—N1—C20—C21	−2.39 (17)	C18—C2—C3—O1	72.0 (2)
N2—C21—C20—O2	107.37 (14)	C1—C2—C3—C4	14.4 (2)
N2—C21—C20—N1	−72.01 (15)	C18—C2—C3—C4	−108.69 (17)
C20—N1—C1—C6	106.90 (13)	C19—C4—C3—O1	−21.7 (3)
C5—N1—C1—C6	−81.41 (14)	C5—C4—C3—O1	−148.50 (19)
C20—N1—C1—C2	−121.17 (12)	C19—C4—C3—C2	158.92 (17)
C5—N1—C1—C2	50.52 (15)	C5—C4—C3—C2	32.2 (2)
C26—N3—C23—C22	113.00 (16)	C17—C12—C13—C14	−1.1 (2)
C24—N3—C23—C22	−54.22 (17)	C5—C12—C13—C14	−178.29 (15)
N1—C1—C6—C11	116.25 (15)	C21—N2—C25—C24	−179.06 (11)
C2—C1—C6—C11	−11.1 (2)	C22—N2—C25—C24	59.52 (15)
N1—C1—C6—C7	−64.17 (16)	C26—N3—C24—C25	−112.15 (15)
C2—C1—C6—C7	168.49 (12)	C23—N3—C24—C25	55.93 (17)
N1—C1—C2—C3	−54.18 (16)	N2—C25—C24—N3	−57.96 (16)
C6—C1—C2—C3	74.44 (17)	C6—C7—C8—C9	0.1 (2)
N1—C1—C2—C18	66.89 (16)	C7—C8—C9—C10	0.1 (2)
C6—C1—C2—C18	−164.50 (13)	C13—C12—C17—C16	1.9 (2)
C11—C6—C7—C8	−0.8 (2)	C5—C12—C17—C16	179.12 (15)
C1—C6—C7—C8	179.55 (13)	C27—O4—C26—O3	−6.8 (2)
C20—N1—C5—C12	−69.90 (15)	C27—O4—C26—N3	173.41 (13)
C1—N1—C5—C12	118.85 (13)	C23—N3—C26—O3	−175.88 (16)
C20—N1—C5—C4	166.64 (12)	C24—N3—C26—O3	−9.4 (2)
C1—N1—C5—C4	−4.61 (16)	C23—N3—C26—O4	3.9 (2)
C13—C12—C5—N1	−54.40 (18)	C24—N3—C26—O4	170.36 (13)
C17—C12—C5—N1	128.50 (14)	C8—C9—C10—C11	0.4 (3)
C13—C12—C5—C4	70.92 (17)	C6—C11—C10—C9	−1.1 (3)
C17—C12—C5—C4	−106.18 (15)	C26—O4—C27—C28	−88.67 (19)
N1—C5—C4—C3	−37.21 (18)	C12—C17—C16—C15	−1.2 (3)
C12—C5—C4—C3	−162.89 (13)	C12—C13—C14—C15	−0.4 (3)
N1—C5—C4—C19	−164.62 (14)	C13—C14—C15—C16	1.1 (3)
C12—C5—C4—C19	69.69 (17)	C17—C16—C15—C14	−0.4 (3)
C21—N2—C22—C23	−178.42 (11)		

### Hydrogen-bond geometry (Å, °)

**Table:**

*D*—H···*A*	*D*—H	H···*A*	*D*···*A*	*D*—H···*A*
C5—H5···N2	0.98	2.51	3.1788 (17)	125
C8—H8···O2^i^	0.93	2.54	3.4406 (19)	162
C24—H24A···O1^ii^	0.97	2.56	3.520 (2)	169

Symmetry codes: (i) −*x*, −*y*, −*z*+1; (ii) −*x*+1, −*y*, −*z*.
